# The Use of a Transforaminal Lumbar Interbody Fusion (TLIF) Cage Following Inadequate Access to the Disc Space During an Anterior Lumbar Interbody Fusion Procedure

**DOI:** 10.7759/cureus.22792

**Published:** 2022-03-03

**Authors:** Malik Obeidallah, Mousa K Hamad, Ryan Holland, Antonios Mammis

**Affiliations:** 1 Neurological Surgery, Montefiore Medical Center, Moses Campus, Bronx, USA; 2 Neurological Surgery, New York University Grossman School of Medicine, New York City, USA

**Keywords:** lumbosacral fusion, alif, tlif, anterior decompression, lumbar spondylolisthesis

## Abstract

Non-specific lower back pain caused by degenerative lumbar disease, such as disc and facet joint degeneration or spondylolisthesis, significantly impairs quality of life of patients and is associated with higher pain scores and reduced function. Patients that fail to respond to conservative treatment may require surgical intervention, such as lumbar interbody fusion (LIF). Compared to other approaches, an anterior approach to lumbar interbody fusion (ALIF) has advantages regarding efficacy of fusion, visualization of relevant anatomy, and a larger allowable size of the interbody fusion device. An anterior approach’s main biomechanical advantage includes the ability to restore sagittal alignment, achieve indirect decompression, and provide increased anterior column support. Complications of anterior interbody fusion are mostly approach related and include vascular injury or visceral injury. However, the anterior anatomy can make the placement of an interbody device challenging. In the case reported here, an ALIF procedure was complicated by immobile iliac vessels leaving a small window to place the interbody cage. Continuing with the anterior approach was opted, but with the oblique placement of a cage traditionally used in transforaminal lumbar interbody fusion (TLIF) procedures.

## Introduction

There are several approaches for lumbar interbody fusions with their own benefits and possible complications [1]. The anterior approach is commonly used for the treatment of degenerative disc disease, spondylolisthesis, tumor, infection, and fracture [2]. Anterior lumbar interbody fusion (ALIF) restores lumbar lordosis and achieves coronal and sagittal balance. This is particularly helpful because loss of physiological lordosis and sagittal imbalance is a potential cause of lumbar spine pain [3]. An anterior approach to the lumbar spine allows for direct visualization and access to the anterior column, thus making it easier to perform a complete discectomy or place a large interbody fusion device [3]. Disadvantages of anterior approaches relate to the anatomical structures encountered during surgery [4,5]. As compared to other approaches such as posterior lumbar interbody fusion, medical complications in ALIF reflect risks intrinsic to mobilization of abdominal vasculature resulting in neurovascular complications that include bleeding and thrombotic events [6,7]. This case study describes a "bailout" technique that may be used if the surgeon cannot adequately access the disc space.

## Case presentation

**Figure 1:**
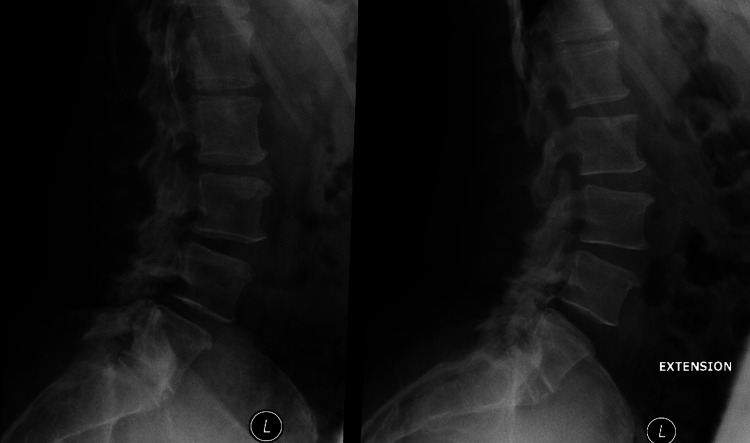
Preoperative flexion and extension of lumbar spine (Left image) Lateral flexion x-ray of the lumbar spine with grade 1 spondylolisthesis that reduces with extension (right image).

The patient was a 52-year-old female. She had a past medical history of chronic fatigue syndrome and presented with chronic back pain. Lumbar spine flexion-extension x-rays showed dynamic instability at L4-5 due to degenerative spondylolisthesis (Figure 1). After an unsuccessful trial of conservative management, the decision was made to perform an ALIF at L4-5.

Exposure at the L4-5 disc space was confirmed in a small window between the Iliac vessels. However, despite the use of vascular surgery, it was difficult to mobilize these vessels and the window could not have been enlarged. Due to this limited access, the decision was made to proceed with the interbody fusion using a Latis® (Globus Medical, Audubon, PA) interbody cage from an oblique approach. This cage was designed to be used in a transforaminal lumbar interbody fusion (TLIF) procedure. Serial disc shavers from 9 mm up to 11 mm were used for the discectomy and the superior and inferior end plates of L4 and L5 were prepped with curettes. Infuse® (Medtronic Inc., Minneapolis, MN) and Actifuse (Baxter International Inc., Deerfield, IL) was packed into a lattice 10 x 32 x 10 mm 8° lordotic cage. The Infuse bone graft consists of recombinant human bone morphogenetic protein-2 (rhBMP-2) and bovine type 1 collagen. Actifuse consists of silicate-substituted calcium phosphate and is a bone void filler intended to fill gaps in the structure of bone. The cage was malleted into position and expanded to a 100% expansion.

**Figure 2:**
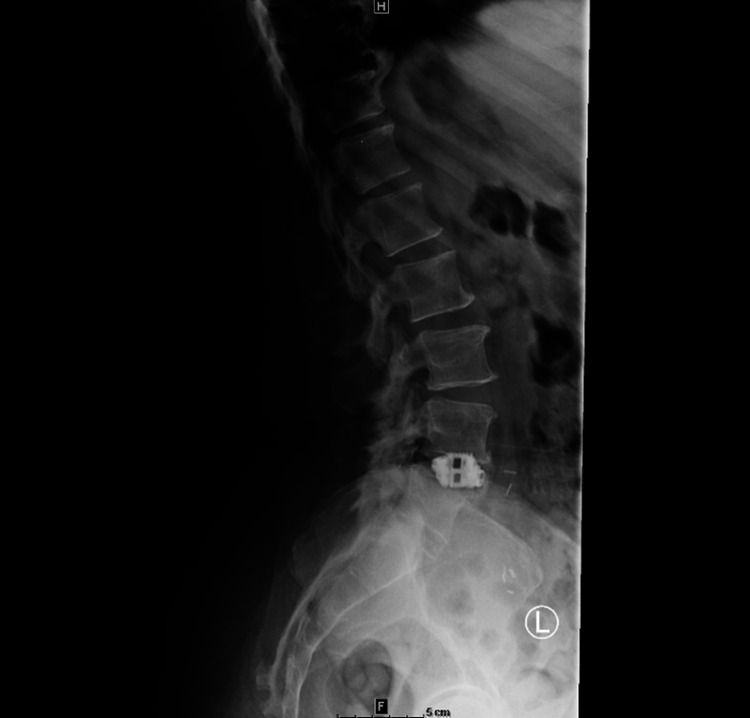
Postoperative lateral view at three months

The postoperative period was uncomplicated, and the patient reported a decrease in the intensity of her usual pain. Monthly x-rays were obtained. At the three-month follow-up, the patient stated the pain was significantly better and was very pleased with the progress (Figure 2). Lumbar spine series confirmed that the interbody spacer at the L4-5 level was unchanged since the surgery. The unchanged spacer and reduction in the patient's pain indicated adequate fusion.

## Discussion

When using an anterior approach, skeletonizing and mobilizing the distal abdominal aorta and iliac vessels places patients at risk of iatrogenic injury [3]. Differences in approach can also influence what graft can be inserted into the interbody space. Recombinant human BMP has been approved by the US Food and Drug Administration (FDA) for use in ALIF procedures with a titanium metallic cage. However, off-label application is widely used in several cervical and lumbar interventions [8,9]. Spinal fusion using an anterior approach has an advantage over other approaches with regard to fusion potential and ability to more safely use rhBMP, and the relatively large osseous surface area means a larger interbody device can be used [3]. The Latis was chosen simply due to its availability at the time of the operation. However, using a larger interbody device can become problematic in cases such as the one described here when access is limited by the anterior anatomy due to immobile iliac vessels. In these instances, we have demonstrated a safe and effective technique of placing an interbody device conventionally used in TLIF procedures in the anterior interbody space during an ALIF procedure. We believe that placing an expandable TLIF graft anteriorly with or without posterior percutaneous screws is a safe and effective salvage strategy. No posterior screws or instrumentation was used at the time of index surgery, but was planned for at three months postoperatively if there was not solid fusion. This decreases the need for additional anesthesia time and surgical risk associated with pursuing a traditional TLIF approach (minimally invasive or traditional open) in a "bailout" procedure.

## Conclusions

Lumbar spinal fusion is a well-established procedure used to join vertebrae to reduce pain or spinal deformity. An anterior approach to the procedure is particularly effective at fusing the vertebrae. If rhBMP is to be placed in an interbody cage, an anterior approach is also less likely to create ectopic bone formation in the spinal canal. However, in situations like the case presented here, surgeons performing an ALIF may find it difficult to adequately mobilize the iliac vessels to create a window large enough to insert an anterior interbody cage. We have shown in this case report that it is safe and effective to obliquely insert a TLIF-designed cage following an anterior approach. This option allows the surgery to continue without repositioning the patient and attempting an alternative approach.
